# Renal arteriovenous fistulas presenting as painless gross hematuria in a young adult male: A case report

**DOI:** 10.1016/j.radcr.2026.04.051

**Published:** 2026-05-18

**Authors:** Nora Khaled Al Shehri, Majed Ali Sahab, Abdullah Ali Asiri, Abdulelah Saad Alabsi, Abdullah Hassan Muhayya, Ahmed Moustafa Aboaly

**Affiliations:** a Department of Urology, Aseer Health Cluster, Abha, Saudi Arabia; b MIS & Robotic Urology, Tours University, Tours, France; c Department of Urology, Aseer Central Hospital, Abha, Saudi Arabia; d Department of Intensive Care Medicine, Ain Shams University, Cairo, Egypt; e Department of Intensive Care Medicine, Armed Forces Hospital Southern Region (AFHSR), Abha, Saudi Arabia

**Keywords:** Renal arteriovenous fistula, Hematuria, Embolization, Angiography, Vascular anomaly

## Abstract

Renal arteriovenous fistulas (AVFs) are rare vascular abnormalities that may present with gross hematuria. Diagnosis can be challenging when conventional imaging modalities fail to detect subtle or intrarenal vascular lesions. We report a case of a 31-year-old male presenting with painless gross hematuria in whom both computed tomography (CT) and magnetic resonance imaging (MRI) were nondiagnostic. Diagnostic ureteroscopy excluded urothelial malignancy, and subsequent renal angiography revealed a high-flow AVF arising from intrarenal arterial branches. The lesion was successfully treated with superselective coil embolization, resulting in immediate resolution of gross hematuria and preservation of renal function. This case highlights the limitations of cross-sectional imaging and underscores the diagnostic and therapeutic value of angiography in patients with persistent unexplained gross hematuria.

## Introduction

Renal arteriovenous fistulas (AVFs) are rare vascular anomalies, representing less than 1% of all renal vascular lesions. They may be congenital or acquired and can present with hematuria, flank pain, or, in severe cases, high-output cardiac failure depending on the degree of arteriovenous shunting.

Diagnosis may be challenging due to variable imaging findings, particularly when lesions are small or lack a well-defined nidus. Conventional cross-sectional imaging such as CT or MRI may fail to detect subtle vascular abnormalities.

Congenital renal AVFs have been previously described and remain an uncommon source of significant hematuria [1]. Endovascular embolization has become the preferred treatment modality owing to its minimally invasive nature, favorable safety profile, and durable outcomes [[1], [2], [3], [4]].

We report a case of a young adult male presenting with painless gross hematuria due to a renal AVF that was not detected on CT or MRI and was successfully managed with selective transarterial coil embolization.

## Case presentation

A 31-year-old male presented with painless gross hematuria and mild right flank discomfort.

Pharmacological history revealed use of over the counter medication which was a non-selective nonsteroidal anti-inflammatory drug (ibuprofen) for a period of time for tension headache used intermittently prior to presentation.

Laboratory evaluation revealed stable hemoglobin and normal renal function. Coagulation profile was within normal limits.

Urinalysis revealed positive blood on dipstick testing with negative leukocytes and nitrites, and no proteinuria. Microscopic examination showed no significant red blood cells, likely reflecting intermittent bleeding. No crystals or casts were identified, supporting a non-infectious and non-glomerular source of hematuria.

Ultrasound demonstrated echogenic material within the collecting system consistent with intrarenal clot burden. Color Doppler evaluation did not demonstrate abnormal vascularity or turbulent flow.

Contrast-enhanced CT (including arterial and venous phases) and MRI failed to demonstrate early venous opacification, vascular nidus, or any definitive vascular malformation, a limitation described in earlier literature [1].

**Fig. 1:**
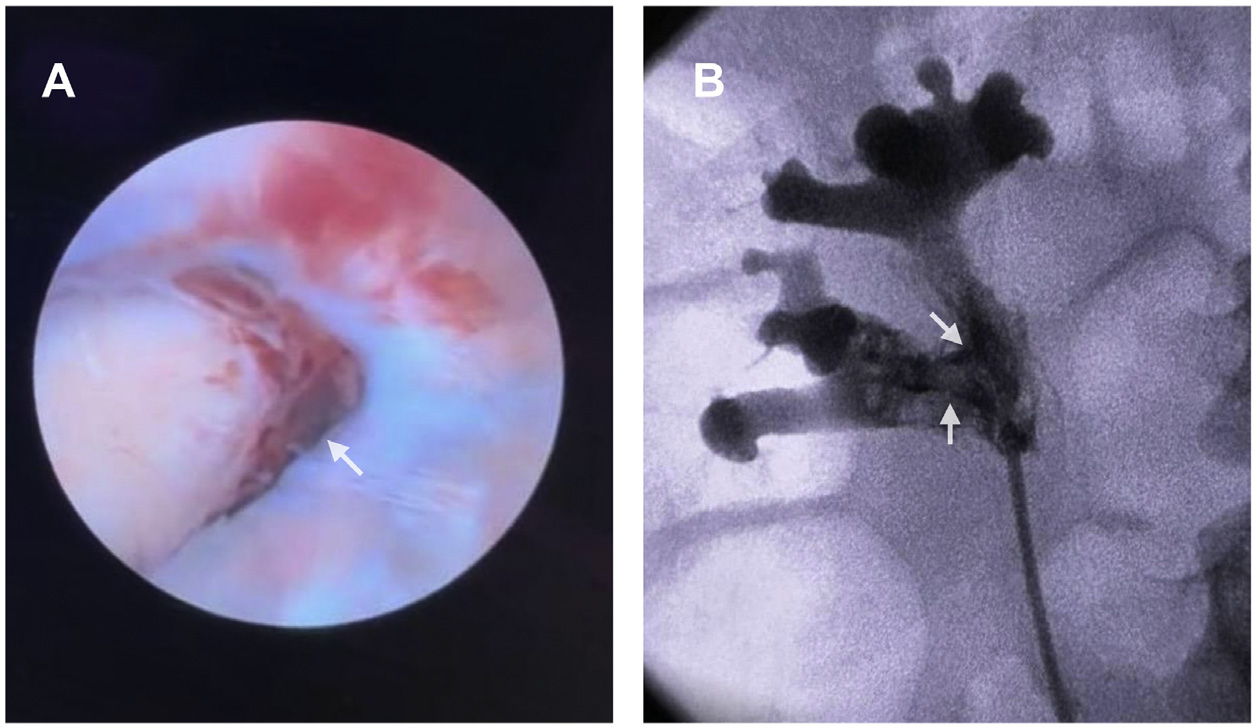
Endoscopic and fluoroscopic evaluation. (A) Ureteroscopic view demonstrating intrarenal clot with focal mucosal bleeding (arrow). (B) Retrograde pyelogram demonstrating contrast opacification with suspected forniceal leak or pyelovenous backflow (arrows).

Diagnostic ureteroscopy was performed to exclude urothelial malignancy given persistent gross hematuria and inconclusive imaging, and revealed intrarenal clot with focal mucosal bleeding (Fig. 1A).

Retrograde pyelography demonstrated contrast opacification with features suggestive of forniceal leak or pyelovenous backflow, likely secondary to increased intrarenal pressure from clot burden (Fig. 1B).

**Fig. 2:**
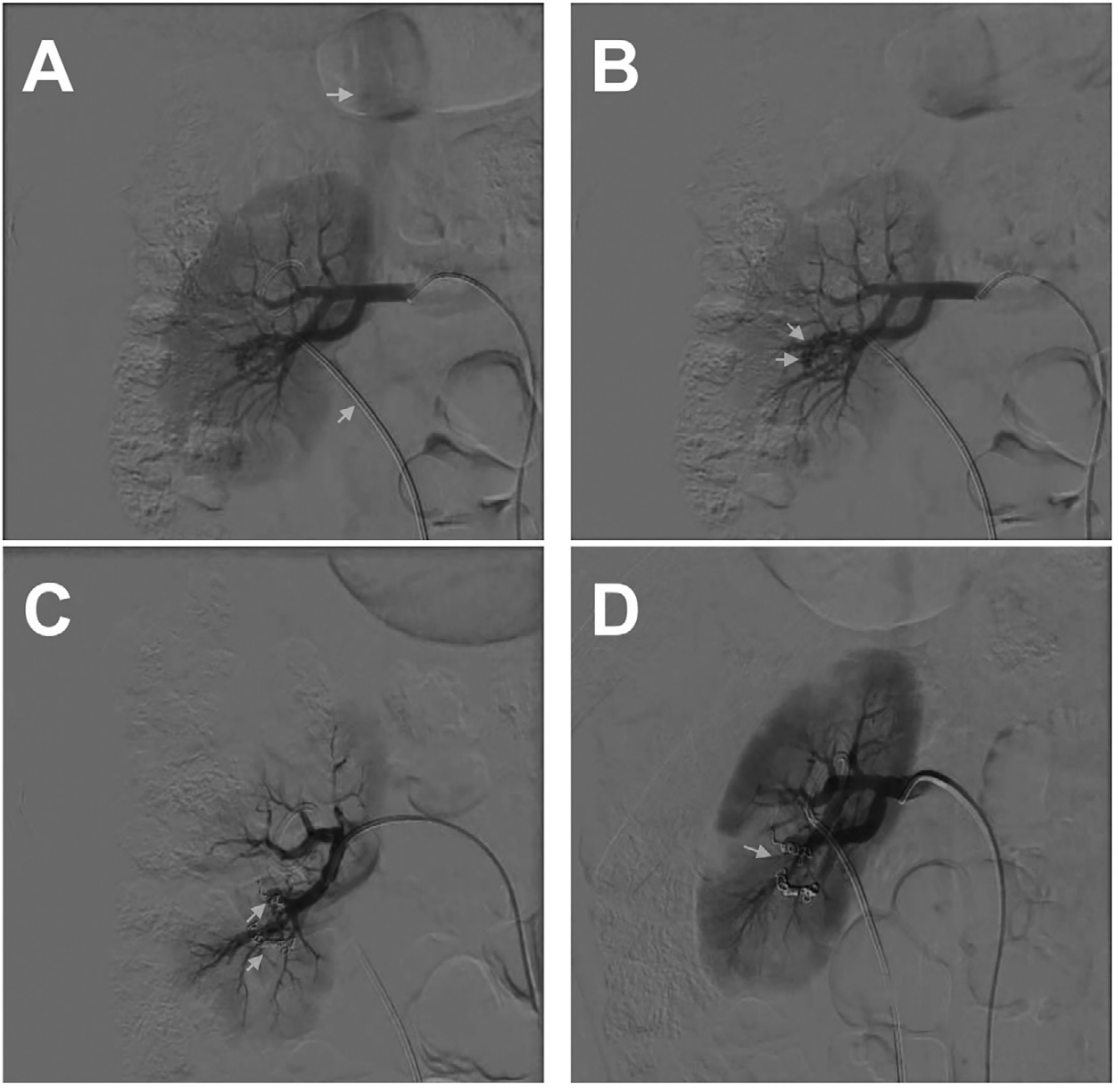
Angiographic findings and endovascular treatment. (A) Initial renal angiogram demonstrating early venous drainage (arrow), consistent with a high-flow arteriovenous fistula. (B) Selective angiogram identifying abnormal arterial feeders and demonstrating fistulous communication prior to embolization (arrows). (C) Fluoroscopic image demonstrating coil deployment within the feeding vessels (arrows). (D) Completion angiogram demonstrating successful occlusion with no residual arteriovenous shunting (arrow).

Given persistent hematuria and inconclusive imaging, a preliminary endourological step was performed with ureteral stent placement to facilitate urinary drainage and optimize visualization. Subsequently, selective renal angiography was undertaken. Initial angiography demonstrated early venous filling consistent with a high-flow arteriovenous fistula (Fig. 2A). Selective angiography identified abnormal arterial feeders arising from distal intrarenal branches of the lower segmental artery (Fig. 2B).

Superselective transarterial embolization was performed via right common femoral artery access using a 6-Fr sheath. A microcatheter was advanced into the feeding branches, and multiple 2 mm fibered stainless steel coils were deployed based on vessel size and flow characteristics (Fig. 2C). Completion angiography demonstrated complete occlusion with no residual early venous drainage (Fig. 2D).

Hematuria resolved immediately following the procedure. Renal function remained stable. No post-embolization syndrome or complications were observed.

At follow-up, serial urinalysis was utilized as a cost-effective and non-invasive tool to monitor for recurrence of hematuria. Findings confirmed complete resolution, with no microscopic or gross hematuria detected, supporting sustained technical and clinical success. No recurrence occurred during three-month follow-up, consistent with previously reported outcomes [3,5].

## Discussion

Renal arteriovenous vascular anomalies include arteriovenous malformations (AVMs), which may be congenital or acquired and are characterized by a nidus of abnormal vascular channels, and arteriovenous fistulas (AVFs), which typically consist of a single direct communication between an artery and a vein, often acquired following trauma or instrumentation without a well-defined nidus. High-flow lesions often present with gross hematuria due to rupture of fragile vessels into the collecting system [1].

Although nonsteroidal anti-inflammatory drugs (NSAIDs) are not implicated in the pathogenesis of renal arteriovenous malformations or fistulae, their inhibitory effect on platelet aggregation is well established [6,7]. By impairing thromboxane A2–mediated platelet function, NSAIDs may increase the risk of bleeding and exacerbate hemorrhagic manifestations [6,7]. In the urological setting, medications affecting hemostasis have been shown to unmask underlying pathology rather than act as primary causes of hematuria [8]. Therefore, NSAID use in our patient may have contributed to the clinical presentation by precipitating or worsening bleeding from a previously silent vascular lesion.

This case highlights an important diagnostic challenge, as both CT and MRI failed to identify the vascular lesion despite ongoing gross hematuria. This underscores the limitations of cross-sectional imaging. This case further highlights the limitation of multiphasic CT and MRI in detecting small or intrarenal AVFs, particularly in the absence of a well-defined nidus or significant early venous opacification. Therefore, angiography remains the gold standard for diagnosis in suspected vascular etiologies.

The angiographic findings in this case demonstrated a high-flow AVF arising from intrarenal arterial branches, with early venous drainage and no discrete nidus, supporting the diagnosis of a fistulous lesion rather than a classical cirsoid AVM.

Endovascular embolization is the first-line treatment for renal AVFs due to its minimally invasive nature and high success rates along with excellent long-term outcomes [[2], [3], [4]]. Superselective coil embolization allows precise occlusion of abnormal vessels while preserving surrounding renal parenchyma. Comparable cases have demonstrated durable symptom resolution and low recurrence rates [5].

This case is particularly instructive as it demonstrates that significant vascular lesions may remain occult on advanced imaging, and that angiography should be considered early in patients with persistent unexplained hematuria and demonstrates the effectiveness of selective coil embolization.

## Conclusion

Renal arteriovenous fistulas are rare causes of gross hematuria. When routine imaging is inconclusive, selective renal angiography remains the gold standard for diagnosis. Endovascular coil embolization is a safe and effective treatment, providing immediate resolution and preservation of renal function.

## Declaration of generative AI and AI-assisted technologies in the manuscript preparation process

During the preparation of this work the authors used Chat GPT(openAI) to assist with language refinement and formatting. After using this tool, the authors reviewed and edited the content as needed and take full responsibility for the content of the published article.

## Patient consent

Written informed consent for publication was obtained from the patient.
